# Smart Buildings: A Comprehensive Systematic Literature Review on Data-Driven Building Management Systems

**DOI:** 10.3390/s24134405

**Published:** 2024-07-07

**Authors:** Adrian Taboada-Orozco, Kokou Yetongnon, Christophe Nicolle

**Affiliations:** 1 K.I.D.S A.I’S, 14 Rue du Golf, 21800 Quetigny, France; adrian.taboada@ciad-lab.fr; 2 CIAD Laboratory, University of Burgundy, 21000 Dijon, France; kokou@u-bourgogne.fr

**Keywords:** building management systems, systematic literature review, buildings, smart buildings, computer science

## Abstract

Buildings are complex structures composed of heterogeneous elements; these require building management systems (BMSs) to dynamically adapt them to occupants’ needs and leverage building resources. The fast growth of information and communication technologies (ICTs) has transformed the BMS field into a multidisciplinary one. Consequently, this has caused several research papers on data-driven solutions to require examination and classification. This paper provides a broad overview of BMS by conducting a systematic literature review (SLR) summarizing current trends in this field. Unlike similar reviews, this SLR provides a rigorous methodology to review current research from a computer science perspective. Therefore, our goal is four-fold: (i) Identify the main topics in the field of building; (ii) Identify the recent data-driven methods; (iii) Understand the BMS’s underlying computing architecture (iv) Understand the features of BMS that contribute to the smartization of buildings. The result synthesizes our findings and provides research directions for further research.

## 1. Introduction

The rapid urbanization of recent decades has significantly challenged our way of cohabiting. With over 60% of the global population now residing in urban areas [1], the strain on city infrastructure and resources has intensified, highlighting the urgent need for scalable technological solutions. Among the most critical areas for innovation is the management of building resources, given that the majority of urban residents spend most of their time indoors [2]. As such, the efficient and intelligent operation of buildings is pivotal to addressing broader sustainability goals such as reducing energy consumption, minimizing pollution, and enhancing the overall quality of life.

A central challenge in the field of Building Management Systems (BMSs) is the fragmentation of information within the literature. Current research often addresses BMS components in isolation, focusing on specific subsystems like heating, lighting, or air quality without integrating these diverse functionalities into a comprehensive framework. This fragmented approach prevents the conception of BMS as a holistic system, leading to inefficiencies and a narrow understanding of the field.

Initially designed to centralize functions like heating [3], BMSs have expanded their scope to include a myriad of functions aimed at enhancing building efficiency across lighting, air quality, and energy use [4,5]. These systems have progressively incorporated more advanced information and communication technologies, subtly transitioning towards what some in the industry refer to as building operating systems (BOSs) [6]. These systems represent a broader, more integrated approach to building management, although their full potential and definition remain fluid within the academic realm. This rapid development has left unsolved questions about BMS that need to be addressed.

This systematic literature review (SLR) paper aims to address the fragmentation of information on BMS in research by providing a systematic and comprehensive overview of BMS technologies from a computer science perspective and highlighting future research directions. Specifically, this review paper delves into the main topics and trends related to BMS research, examines recent data-driven methods applied to BMS, clarifies the underlying computing architecture of recent BMS, and unmasks the vague connotation of smart in the context of BMS [7,8]. The main contributions of this SLR are three-fold: (i) it adopts a holistic approach, synthesizing diverse research on BMS as an interconnected ecosystem; (ii) it clarifies key concepts, trends, and data-driven methods to illuminate the current landscape of BMS research; and (iii) it identifies gaps in the literature and highlights promising avenues for future research.

## 2. Review Methodology

### 2.1. Planning the Review

**Figure 1:**
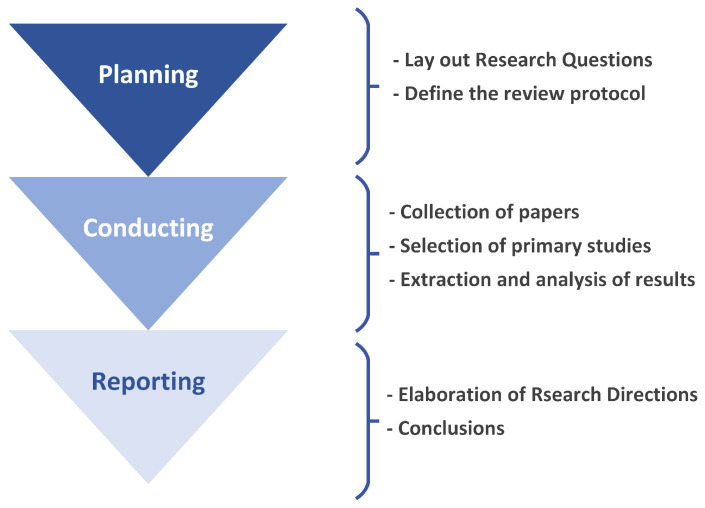
General diagram of the steps of the SLR conducted in this paper.

**Figure 2:**
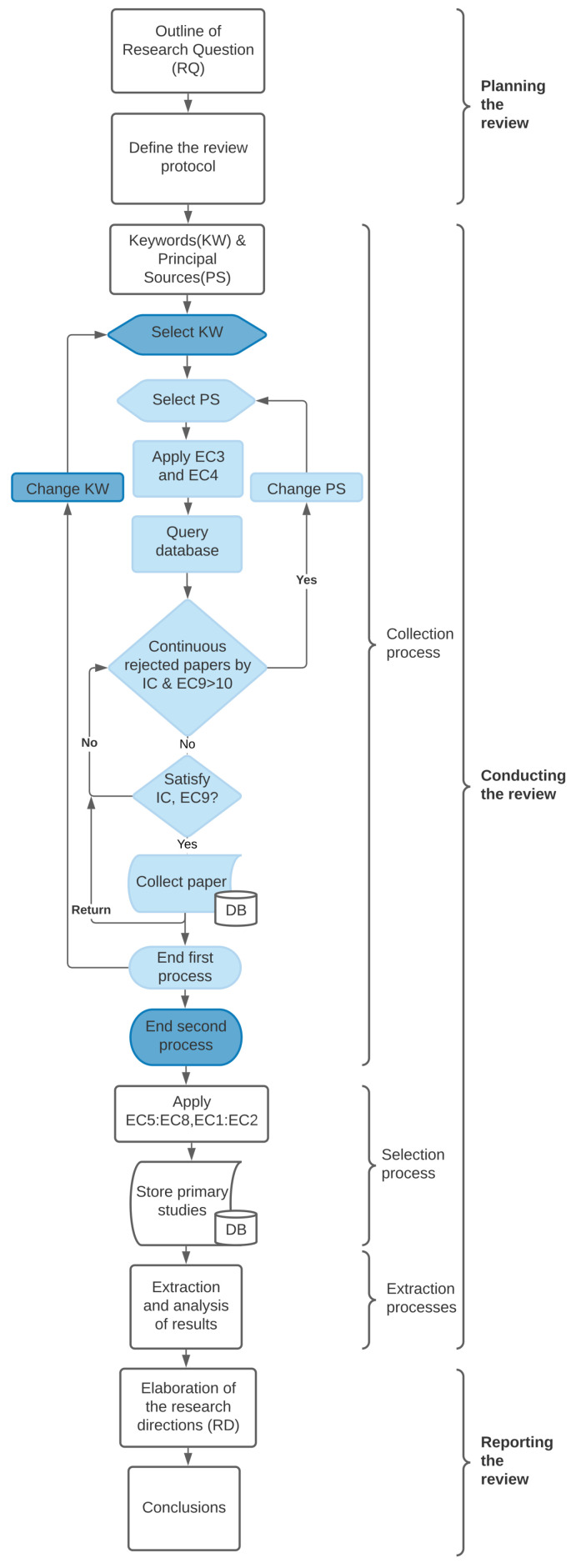
The flow diagram shows the steps followed in the SLR. In the collection process, the dark blue represents the collection process per keyword and nested in light blue is the collection process by principal source. The collection process ends in the second process.

A systematic literature review methodically summarizes research within a specific domain to establish a solid foundation for new studies [9]. With recent technological advancements introducing numerous building management solutions, a systematic exploration and classification of these innovations are essential. Defined by Fink [10] as a “systematic, explicit, and reproducible method”, SLRs aim to consolidate research around a topic and identify gaps for future investigation. Distinct from traditional reviews, SLRs emphasize the transparency and reliability of their evaluation thorough documentation [11]. Petticrew [12] suggests using SLRs when extensive research exists but significant questions remain, a situation evident in the rapidly evolving field of BMS due to ICT integration. This SLR seeks to summarize and highlight developmental needs within BMS. This SLR process includes three phases: planning, conducting, and reporting the Review [9], illustrated in Figure 1 and Figure 2. The planning phase comprises three subsections: defining the research questions (Section 2.2), establishing the review protocol (Section 2.3), and examining similar reviews in the BMS field (Section 2.4) to ensure this SLR’s relevance and novelty. Besides this process, we focus on attaining the Preferred Reporting Items for Systematic reviews and Meta-Analyses (PRISMA) checklist found in the Supplementary Materials, and registered on the Open Science Framework (OSF) https://osf.io/9y3cz/?view_only=83e68120083b428ba877f3ea5cc261ae, accessed on 6 May 2024.

### 2.2. Research Questions

The formulation of research questions is a pivotal component of an SLR and is driven by the gaps identified in prior research [11]. Our questions are developed through an iterative process, influenced by our previous studies on BMS [13,14]. This section outlines the refined research questions that guide our inquiry into the evolving field of BMS.

- RQ1—This question aims to delineate the current scope of BMS by focusing on emerging technologies and operational strategies. Understanding these areas will highlight recent advancements and persistent challenges within the field.

 
**What are the prevailing technological and operational interests and services in the BMS field?**

- RQ2—This inquiry focuses on the ICT tools and methodologies used within BMS to support decision making and optimize building management. Clarifying these methods will contribute to understanding how data-driven approaches are integrated into BMS.

 
**What methods are employed to analyze and utilize data in BMS to enhance operational services?**

- RQ3—By categorizing BMS based on their major features and technological innovations, this question seeks to provide a comprehensive taxonomy of BMS types, aiding stakeholders in selecting appropriate systems.

 
**What are the different types of BMS available, and what are their defining features?**

- RQ4—This question explores the underlying computing frameworks and architectural designs of BMS, particularly focusing on scalability and distribution. Understanding these architectures is crucial for developing systems that are both efficient and adaptable.

 
**How is computing architecture distributed within BMS across different geographical settings?**

- RQ5—This question aims to dissect the elements of BMS that enhance the intelligence and responsiveness of buildings. Defining ‘smartness’ in this context will help in measuring the effectiveness of BMS in improving building operations.

 
**How do BMSs contribute to the smartness of buildings, and what specific features are most impactful?**

### 2.3. Reviewing Protocol

The protocol establishes how the review process is conducted. This subsection is divided into five sections: Section 2.3.1 identifies the source of information for this SLR. Section 2.3.2 describes the querying keywords. Section 2.3.4 describes the criteria when selecting papers. Section 2.3.5 describes the evaluation of the quality of each selected paper. Section 2.3.6 describes the selection criteria before and while searching papers.

#### 2.3.1. Source of Information

To ensure the accuracy and reproducibility of this SLR, we have carefully selected the information sources based on criteria established by [15,16,17]. These criteria include the accessibility of sources, the availability of Boolean operators to refine searches, and the relevance of the sources’ scope to BMS. By applying these criteria, we aim to enhance the precision of the literature search and ensure that the review process is fully reproducible.

Given the multidisciplinary nature of BMS, wherein electrical and electronic engineering and computer science intersect, we prioritize sources that cater to these disciplines while also considering specialized and multidisciplinary databases to maintain a balanced perspective. The selection strategy involves extensively using primary sources, with a provision to resort to auxiliary sources, as detailed in Table 1 [15], should challenges arise in accessing full texts. This approach ensures a comprehensive coverage of BMS literature, addressing both principal and emerging themes in the field.

#### 2.3.2. Keyword Selection

The keyword selection is directly informed by the research questions outlined in this study, ensuring a targeted and relevant search within the BMS field. Keywords such as “Building”, “automation”, “supervision”, and “monitoring” reflect the core themes and objectives from our research questions. These terms help us focus on the specific aspects of BMS we aim to explore, such as technological advancements and operational strategies.

To achieve comprehensive coverage and account for variability in terminology across studies, we also use synonyms like “Building Management Systems” and “Building Automation Systems”. Although these terms are similar, they cater to different research nuances and are crucial for capturing the breadth of literature on BMS.

Boolean operators are employed to refine the searches further, creating precise combinations that exclude irrelevant material.

This strategy ensures that every keyword and search term used is a reflection of the research questions, enhancing the specificity and accuracy of literature review. By systematically connecting the keywords to our research aims, we ensure that the scope of our search is both focused and exhaustive.

The keywords for querying the principal sources are as follows:

- “Building” AND “Management” OR “Automation” AND “Systems” NOT “Review” NOT “Survey” NOT “Demo”
- “Building” AND “Management” OR “Automation” AND “Systems” AND “Control” NOT “Review” NOT “Survey” NOT “Demo”
- “Smart” OR “Intelligent” AND “Building” NOT “Review” NOT “Survey” NOT “Demo”
- “Building” AND “Management” OR “Automation” AND “System” AND “Data-driven” AND “Artificial Intelligence” NOT “Review” NOT “Survey” NOT “Demo”

The quotation marks around, for example, “Building” ensure searches use this exact term. Note: The Boolean operators shown are examples; actual database syntax may vary.

#### 2.3.3. Selection of the Time Frame

**Figure 3:**
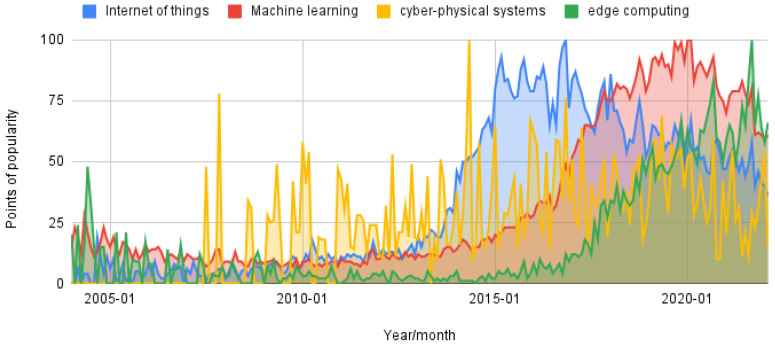
Trends between 2004 and 2020 of some technologies including the Internet of Things, machine learning, cyber-physical systems, and edge computing. Between 2010 and 2021, noticeable the rapid growth in the popularity of these technologies.

The time frame in an SLR depends on the domain of study. There are no absolute wrongs and rights when selecting criteria such as time frame [11]. However, we consider it reasonable and justifiable to focus on the last decade, and take research from 2012 to 2022, but including and considering the relevance of the most recent research (2023, 2024). The rapid growth in fields like the Internet of Things (IoT), learning-based algorithms, edge computing, and cyber-physical systems has been reported by some international indexes like IDC, www.idc.com, accessed on 21 May 2023. These reports refer to the rapid growth and forecast its impact on the economy, society, and technology. A simple query in some open trend analyzers, such as Google Trends, can display the growing interest in these fields. Figure 3, for example, displays the trends from 2004 to 2021.

#### 2.3.4. Inclusion and Exclusion Criteria

This SLR uses clearly defined criteria to filter studies relevant to BMS, guided directly by our research questions [11]. “Inclusion criteria (IC)” identify essential aspects required in studies, while “exclusion criteria (EC)” pinpoint specific reasons to disqualify papers.

- IC1: Studies must primarily focus on buildings systems or other built environments such as houses and parking lots, addressing relevant BMS issues.
- EC1: Studies must examine systems managing multiple sources within a building, excluding those focusing on single smart objects or isolated hardware/software (e.g., smart window, smart door, etc.).
- EC2: The BMS architecture must be explicitly detailed.
- EC3: Only English-language papers are included, ensuring consistency with our keywords.
- EC4: We include studies published from 2012 to 2022, aligning with our need for the most recent research data.
- EC5: We exclude papers that lack comprehensive detail, such as those limited to abstracts or brief descriptions.
- EC6: Review and survey papers are summarized in a specific section and not included for primary analysis.
- EC7: Demo or poster presentations, which typically lack sufficient detail for thorough analysis, are excluded.
- EC8: Extended papers that do not provide new findings but repeat previous studies which are treated as duplicates.
- EC9: Duplicated papers from different databases or multiple versions of the same study are excluded to avoid redundancy.

#### 2.3.5. Quality Criteria

As SLR broadly studies scientific research, evaluating primary studies’ quality is critical [9,18]. Unlike IC and EC, quality criteria (QC) are formulated as questions to assess the scientific value of authors’ research. QC aims to evaluate the quality of the source. In this SLR, each paper of the primary study is validated by QC questions. The method of evaluation is explained in more detail in Section 2.5. The QC questions are shown in Table 2.

#### 2.3.6. Selection Strategy and Checklist Procedure

The search strategy is designed to minimize bias and enhance the reproducibility of results, structured into two key steps:

*Pre-searching step*

Prior to each search, we clear all cookies and cache to reset the search environment. This step ensures that our results are not influenced by previous activities, following recommendations from [16] to secure unbiased search outcomes.

*Searching step*

We employ the test–retest method to validate the consistency of our search results, conducting initial searches in October 2020 and repeating them in April 2022. This approach helps us monitor the stability of search outcomes over time. Two researchers independently perform each search, with a third acting as a mediator to resolve any discrepancies, thereby reducing potential bias. The slight variations observed in the search result ordering are attributed to the ongoing updates and natural growth of the database contents [15], underscoring the dynamic nature of digital libraries.

These steps, combined with our stringent inclusion and exclusion criteria, significantly lower the risk of bias, supporting the integrity and reproducibility of this SLR.

### 2.4. Similar Reviews

Building management systems (BMSs) span multiple disciplines including computer science, hardware and software engineering, telecommunications, and networking. While previous studies have explored individual BMS components and their communication protocols [19], offered historical overviews [3], emphasized control systems for HVAC efficiency [20], and investigated modern BMS development across different scales [4], our review takes a broader and more integrative approach. Unlike reviews focusing on single topics [21,22] or technical details [23], our systematic review covers the entire field to uncover hidden relationships. Our target audience includes researchers and practitioners in computer science and building automation, contrasting with studies aimed at urban planners and designers [24]. In contrast to research centered on building planning and maintenance [25], our focus is on the operational phase of BMS, illustrating how data flows and are exploited within existing architectures. In summary, our review highlights the transformative integration of different subfields in BMS, emphasizing cross-domain data exploitation and the diversification of data sources.

### 2.5. Conducting the Review

This phase consists of three processes: Collection of papers, selection of the primary studies, and extraction and analysis of results, as shown in Figure 1. Therefore, we follow the protocol established in the “Planning the review” phase.

*Collection process*

We conducted our paper collection from principal source databases, detailed in Table 1, using predefined keywords. The search was configured to only include English papers from the specified date range (EC3 and EC4) and continued until ten consecutive papers were excluded, indicating saturation from that source.

**Figure 4:**
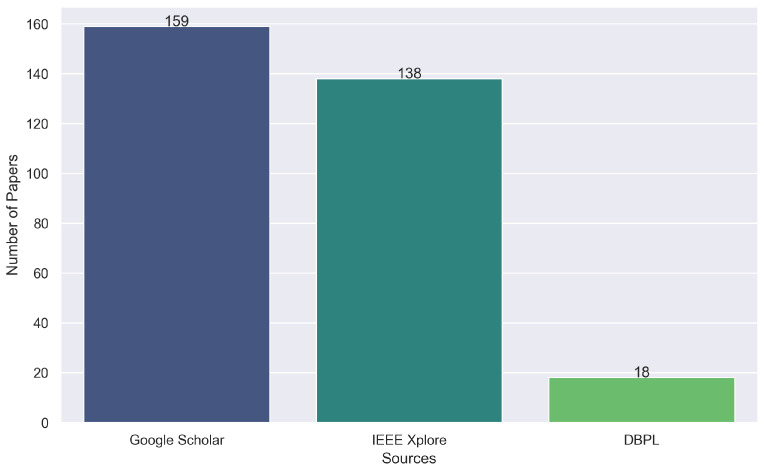
Number of collected papers per principal source.

Initial screening involved applying inclusion criteria (IC) focused on ‘Building’ as the main subject and excluding duplicates (EC9), resulting in a collection of 215 papers. The process was repeated across all sources and keywords until no further keywords remained. The number of papers collected from each source is displayed in Figure 4.

*Selection process*

**Figure 5:**
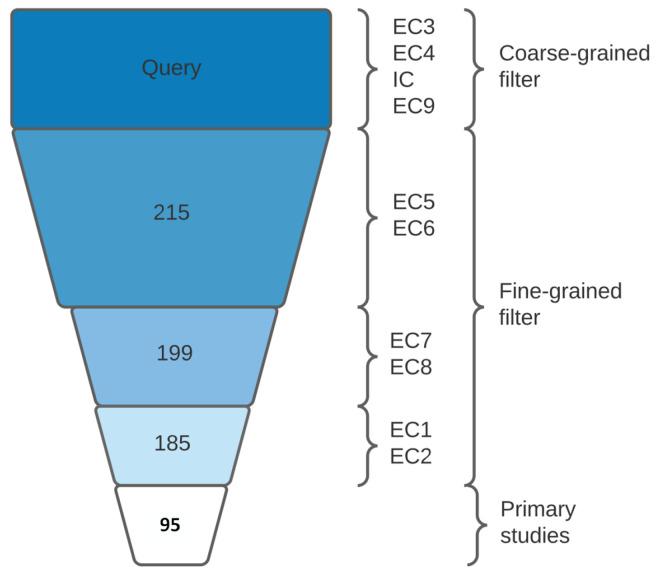
A total number of papers after the application of each inclusion and exclusion criteria.

**Figure 6:**
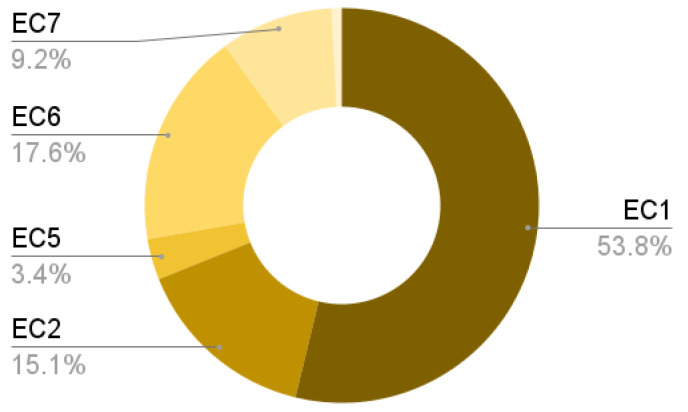
Impact of each EC in the selection process.

We applied EC5, EC6, EC7, EC8, EC1, and EC2 in the selection process. The exclusion criteria in this process serve as a fine-grained filter to obtain the primary studies. A general panorama of the amount of excluded papers in the collection and selection process is shown in Figure 5. Additionally, Figure 6 displays the EC and IC impact (in percentage) on the 215-papers database.

The result of both aforementioned processes is 95 papers, which shape the primary studies [26,27,28,29,30,31,32,33,34,35,36,37,38,39,40,41,42,43,44,45,46,47,48,49,50,51,52,53,54,55,56,57,58,59,60,61,62,63,64,65,66,67,68,69,70,71,72,73,74,75,76,77,78,79,80,81,82,83,84,85,86,87,88,89,90,91,92,93,94,95,96,97,98,99,100,101,102,103,104,105,106,107,108,109,110].

**Figure 7:**
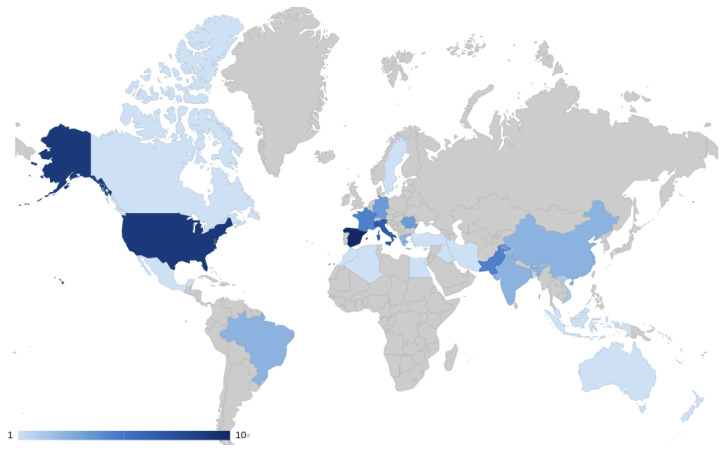
Primary studies in a world map.

**Figure 8:**
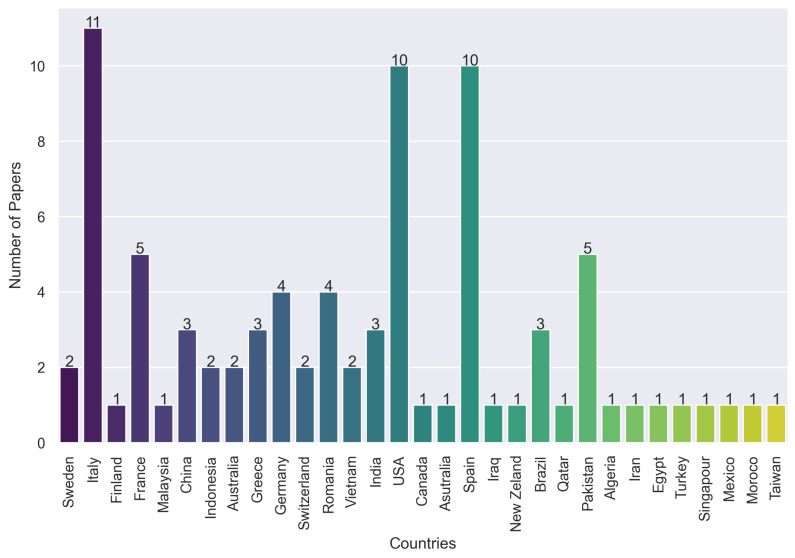
Number of papers by country.

After identifying the primary studies, we analyzed their characteristics including publication year, type, and geographic origin. From 2012 to 2022, it is noticeable that most of the papers come from 2017. The demographic analysis indicates Italy, the USA, and Spain as leading contributors in this field, as illustrated in Figure 7 and Figure 8.

**Figure 9:**
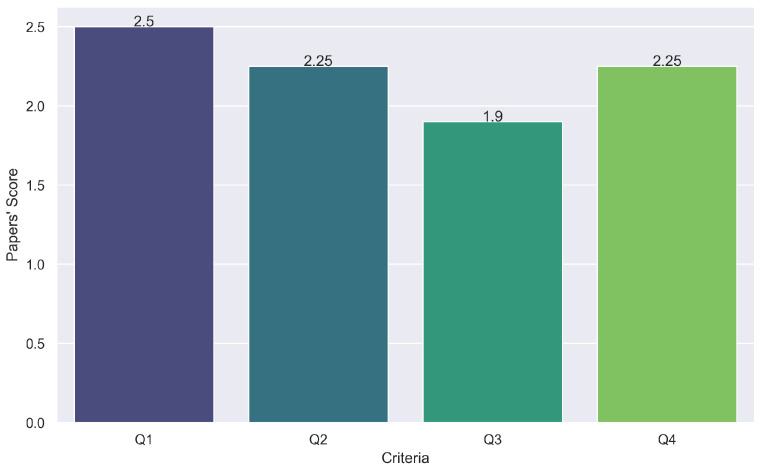
The average score of quality criteria for the primary studies.

To assess the quality of these studies, we assigned each paper a score based on the quality criteria (QC): 3 points for fully met (yes), 2 for partially met, and 1 for not met (no). This scoring, detailed in Figure 9, followed established guidelines by [18,111] to ensure consistency and reliability in our statistical evaluation.

*Extraction, analysis, and synthesis of results*

We extensively reviewed each paper in extracting and analyzing the results process. To avoid disrupting the goal of this SLR (report), we have not established a template or a classification strategy a priori. Therefore, we separately compiled each RQ’s finding, then analyzed and identified patterns per compilation.

Our template consists of a table containing columns of, paper name, year of publication, journal, and short description. For each research question, each author selected the following information:

Upon classifying each primary study into a template table, authors individually interpreted the results, extracting patterns for each research question. These interpretations were organized into separate sub-tables, facilitating focused analysis. Scheduled meetings were held to collectively present and discuss the results, ensuring consensus on the classification of identified patterns. To mitigate bias, the filled template table was cross-checked by presenting it for validation, doubling as a safeguard against potential inaccuracies. Additionally, during the synthesis phase, measures were taken to address potential biases. Firstly, the synthesis process was conducted by multiple researchers independently to minimize individual biases. Any discrepancies or disagreements were resolved through discussion and consensus-building among the research team. Secondly, a thorough and systematic approach was adopted in synthesizing the results, ensuring all relevant data were considered and integrated impartially. By adhering to these rigorous methods, our study aimed to uphold the highest standards of objectivity and integrity in the synthesis of results.

## 3. Results

### 3.1. Identification of Fields of Interest, Issues, and Data Exploitation Methods (RQ1, RQ2)

Merging the findings of RQ1 and RQ2 provides a holistic understanding of how BMS are evolving in response to advances in technology and user demands. RQ1 identifies the key areas of interest within BMS, segmenting them into service-oriented and architecture-oriented studies. This segmentation is crucial as it lays the foundational understanding of the scope and methodologies prevalent in the current research landscape of BMS.

RQ2, on the other hand, delves into the specific application of ICT components like AI within these areas, exploring how they are leveraged to enhance BMS functionalities. By investigating how these technologies are integrated into both the service and architectural frameworks identified in RQ1, RQ2 provides insights into the practical implications of technological advancements in BMS.

Fusing the outcomes of these research questions allows us to see the direct relationships and impacts of ICT innovations on BMS practices. It illustrates how advancements in technology are not only enhancing specific BMS services but also influencing the broader architectural strategies that define the system as a whole. This integrated view is essential for developing a comprehensive understanding of the state and trajectory of BMS research, highlighting areas where further technological integration can significantly improve system efficiency and effectiveness.

#### 3.1.1. BMS-Service-Oriented Studies

This subsection reports five fields of interest focusing on BMS services toward users.

***Energy consumption optimization*** Building energy usage is by far the most essential subject addressed by researchers. This area of study focuses on determining the source of energy waste (e.g., inhabitants) to optimize the management of subsystems such as HVAC.

- Occupant behavior—The authors [29,31,59,60,66,72,78] propose to center on occupants’ activities to construct behavioral models, and improve occupants’ decision making.

 The primary goal of these studies is to identify and model occupants’ energy wastage patterns to optimize building energy use. These models are effective in scenarios like lighting control and thermal regulation [72,78]. However, discrepancies between model predictions and actual behaviors often lead to occupant dissatisfaction, particularly with automated systems like lighting, highlighting the need for more accurate modeling.
 Addressing the challenge of aligning behavioral models with actual occupant behavior, researchers have explored several strategies to enhance model accuracy by diversifying data sources. For instance, Garcia [59] utilizes contextual data from a serious game platform to analyze and communicate the effectiveness of resource use to occupants, thus encouraging more efficient behaviors. Additionally, Khalid [72] enhances predictions by developing an activity-aware system that monitors and analyzes usage patterns of electrical devices to forecast future activities. This approach not only refines the model’s accuracy but also supports smarter energy scheduling aligned with smart grid capabilities.
 To refine the accuracy of behavioral models, researchers employ a range of sophisticated algorithms. These include using diverse algorithms for energy optimization [29,31], employing collaborative learning techniques to integrate contextual data [59], and utilizing predefined scheduling models [66]. Additionally, hybrid approaches that combine bacterial foraging with genetic algorithms [72], as well as fuzzy logic systems [60], are used to handle multiple variables and improve decision-making accuracy in BMS.

- Localization and occupancy—Research underscores that the localization and occupancy of building spaces significantly influence energy consumption, challenging the traditional focus solely on the usage of objects within buildings [63,65,67,71,74,77,79,80,81,89,90,91,105,106,107]. Unlike usage models that monitor device interaction, occupancy and localization models focus on how space utilization affects energy needs. A primary challenge in this area is accurately detecting occupancy without compromising occupant privacy. This issue extends beyond the mere accuracy of sensors; it encompasses the need to consider the characteristics and intended use of each room, such as size and function. For instance, Huang et al. utilized audio signals to estimate a room’s occupancy and purpose, enabling the context-sensitive control of HVAC systems to match the detected patterns of use-activated heating or cooling based on short-term or long-term occupancy [79]. Similarly, Elkhoukhi et al. focused on the dynamic behaviors of occupants, using these data to enhance the energy efficiency of building operations [91]. To effectively leverage occupancy data, various sophisticated techniques are employed to enhance building energy management. Knowledge bases aggregate and process occupancy data, applying predefined rules to deduce room usage patterns and optimize energy distribution according to actual needs [63,77,90,105,107]. Additionally, the K-nearest neighbor algorithm, combined with Kalman filters, is used to improve the accuracy of spatial classification, ensuring that energy management systems are finely tuned to the specific layouts of a building [71,91]. Moreover, fuzzy logic is applied to determine the functionality of different spaces, enabling more nuanced and adaptive energy management strategies [80]. Together, these approaches contribute to a more efficient, responsive, and context-aware energy management system.
- Energy demand-based solution. The authors in [32,49,50,55,56,75,85,97,99,102] proposed using energy rate costs to operate building systems more efficiently, treating buildings as dynamic energy reservoirs that both consume and store energy. For instance, boilers can store thermal energy, optimizing the timing of electricity use. Occupants are now seen as “prosumers”, both consuming and producing energy, complicating the balance of fluctuating energy sources like solar panels. To manage these variations, strategies include integrating thermal and occupancy models with predictive algorithms [32,50,56,75] to adapt energy use to real-time pricing, enhancing both efficiency and cost-effectiveness.

***Healthcare assistance***

The authors [42,43] recommend using BMS to support physically fragile individuals, such as the elderly and rehabilitation patients, by automating tasks like monitoring CO_2_ levels and opening windows. These systems offer a cost-effective alternative to expensive wearable medical devices. However, challenges include ensuring the autonomy of BMS subsystems and maintaining the privacy of highly personal data, as these systems need to adapt to individual behaviors without compromising confidentiality.

The author [42] suggests using data abstraction and a centralized architecture to simplify data management and enhance system responsiveness. This approach aims to provide personalized support efficiently while addressing privacy concerns inherent in personal data handling.

***Indoor navigation***

The authors [54,76,84,87,92,101,110] propose BMS systems to solve indoor displacement problems, which are usually present in large buildings. These systems collect positioning information from sensors such as GPS, infrared, and microphones. Some applications of these studies are the construction of evacuation routes and assisted guidance for disabled people. The most addressed issues in this type of service are the construction of pathways and the distribution of this information. Path-finding algorithms (e.g., Dijkstra) and enhanced visualization tools (e.g., augmented reality) are examples of solutions. The unresolved question is how to integrate data from other sources such as occupancy and accessibility (limitation of access) into the pathway construction.

***Occupants well-being***

The main goal of BMS is to provide wellness to dwellers with cost-effective means. This SLR has identified wellness unfolded into air quality [57,98], thermal comfort [53], and illumination comfort [108].

The main issue in addressing air quality is identifying the pattern of gas dispersion into the building’s dependencies and creating strategies to deal with it. Moreover, thermal comfort’s main issue is orchestrating the HVAC system according to different criteria.

To improve air quality, some solutions like in [57] apply neural networks to estimate the time HVAC to clear away CO_2_ excess. Concerning thermal comfort, some solutions include centering the criteria on activating/deactivating HVAC based on the dweller’s routine [53]. The author [52] has used the thermal satisfaction known as predicted mean vote (PVM) as criteria, based on humidity, temperature, and peoples’ metabolic and mechanical work. Research into thermal comfort is mostly about finding the best combination of events that trigger HVAC systems. For illumination proposes, solutions include fuzzy logic [108] that combines different criteria (input) to make inferences over linguistic rules and find the most optimal output. In this case, the output is the activation of the lights.

#### 3.1.2. BMS-Architecture-Oriented Studies

This subsection reports the studies oriented towards the inner features of the BMS architecture. These fields of interest focus on improving the management of sensors and actuators data, devices data, decision-making, and adaptability. This SLR classifies studies as follows:

***Heterogeneity of the data source***

In the papers, researchers address heterogeneity at two levels: data heterogeneity and device heterogeneity, as follows:

- Data heterogeneity—The heterogeneity of data refers to the format and type of data. An example of the heterogeneity of data is the case of sensors. They can measure innumerable physical phenomena resulting in large datasets. Some of these datasets (e.g., temperature measurements) can contain different unities (e.g., Fahrenheit ) and instrumental precision. In a more complex scenario, the data gathered by BMS may contain data types (e.g., string, integers, text, etc.)

 The authors [26,27,28,35,36,62] identify the heterogeneity of data as an issue that leads to confusion on the applicability of services. The non-uniform abstraction of data from sensors and actuators harms creating applications, especially in the high human and building interaction field.

- Devices heterogeneity—The heterogeneity of the devices’ features refers to the diversity of devices’ features, such as communication protocols, power levels, processing, and storage. The authors [26,27,28,35,36,62] consider the devices (intended appliances) of BMS as subsystems (multi-purpose appliances) and BMS as an ecosystem of them. The heterogeneity of devices increases the operational complexity of BMSs.

 In contrast with data heterogeneity, the mismatch in technical features is strongly linked to the operation of BMS rather than the services it can offer. The operativity of BMS relies upon its exchange of information and data flow between subsystems. The authors in [30,34] associate the heterogeneity of devices’ features with other issues like the siloing of data and blocking interoperability.

The solution for both types of heterogeneity seems to be a unifying descriptive model of features and roles. The authors propose these solutions [26,27,28,35,36,62].

Describing the device’s functional and feature information is essential to mapping (search) data and knowing the initial conditions under which the data were collected (e.g., origin, time, type, precision).

This meta-information might be useful for precisely conducting actuator instructions and improving the decision-making algorithms. This is the case of the work of the author [34], who applies XML to construct a tag model of the functionality of devices. Thus, devices are embedded with this information and requested when needed. Some other authors, like [28], apply more complex tools such as ontologies (BMSont) to define the functionalities and features of these devices. The advantage of more complex descriptive models, such as ontologies, is the association between the concepts and their aggregated context.

Behind descriptive models, researchers have discordance about what to describe. For instance, the authors in [26,27] focus on describing the functionalities of devices, while others, such as [28], describe the objects themselves.

Very few authors address partial models that include the association of the spatial scope of sensors and actuators (e.g., range of motion detectors in rooms) in a machine-readable way. Another point missing in these models is the lack of description in terms of the capability of devices (e.g., processing and storage) which can be helpful in distributing tasks all along with buildings.

***Big-data management***

Nowadays, data have become valuable to understand and customize solutions for users. Buildings are an excellent example of Big Data. These structures are rich in information and are equipped with various devices that constantly collect and produce data. This SLR reports a constant interest of researchers [30,34,39,40,52,57,58,63,71,72,83,94,96,102] in dealing with Buildings’ big data.

The authors in [40,77,102] identify the scalability of databases as an issue related to big datasets. The scalability of classical relational databases is an issue due to reliability (consistency and integrity) and the allowance of transactions. This is an issue for BMS due to its constant transactions between databases in different computing layers. Moreover, the authors [30,52,57,94] address the response time of BMS as a big data issue. When real-time action is needed, obtaining and processing the information in large datasets is challenging. Another issue in this field is the consistency of data [58,63,77]. The devices in the building are constantly producing data. However, because these data are asynchronous, quickly generated, and voluminous, the information may not be concise or clean. This fact makes the large datasets incomplete and imprecise, which might create uncertainty about the awareness at those missing times.

***Decision-making-related issues***

Decentralized decision-making is employed in some approaches [31,32,37,38,41,47,59]. These aim to provide a more effective and quicker answer to BMS users’ demands. Although decentralization seems to show the optimization of BMS response, there seems to be a need for spatiotemporal information. For instance, the authors [32,38,41,59,61,100] studied the orchestration of decisions in BMS. These propose to provide spatial context to BMS devices. The authors imply that this information could be necessary to improve accuracy when orchestrating decisions for many devices. Similarly, the authors in [67,75] suggest that spatial information can be helpful even to estimate the number of sensors or actuators in the buildings to avoid blind spots and maximize resources.

Decentralized decision making seems to be even more challenging. The BMS manages subsystems that make individual decisions, and these can influence the expected outcome of each of them. This implies that orchestrating subsystems might drive the uncoordinated operation of the actuators controlled by these subsystems. Instead, it seems advisable to unify subsystems, data, and actuators in a single BMS and not in multiple single-minded BMSs.

***Adaptability***

The authors [42,44,74,78,90] point out that the complexity of BMS relies on its capability of adaption. Since re-adapting BMS requires one to change its rigid original scope, this is complicated when there is no interoperability between the sub-systems that are part of BMS.

Some authors [45,46,60,80,93,95,101] propose the high-level abstraction of building information (sensors’ data, device data, and information form the building structure) that simplifies the operation of BMS, allowing the traceability of events.

**Figure 10:**
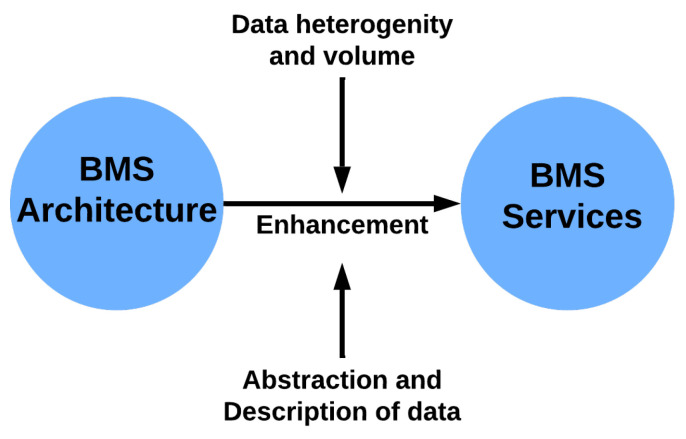
The relation between BMS architecture and BMS services expresses an enhancement when data sources are increased, abstracted, and represented.

This section classifies the areas of interest, challenges, and available solutions. Table 3 summarizes the RQ1 outcomes, and Figure 10 illustrates the relation between BMS architecture and services.

The systematic review of building management systems (BMSs) identified several classes of data-driven methods that enhance system performance and service delivery, as detailed in Table 4. These methods are categorized into four main classes: Machine learning, deep learning, semantics and ontology reasoning, and various algorithms. Within the machine learning class, techniques such as anomaly detection (isolation forest), artificial neural networks (ANNs), k-nearest neighbor (k-NN), support vector machines (SVMs), and reinforcement learning (RL) are employed to predict the energy usage, detection faults, and enhance security. The deep learning class includes advanced models like CNN and LSTM, faster R-CNN, and deep reinforcement learning, which are pivotal for real-time occupancy detection, energy consumption prediction, and optimizing control policies. Semantics and ontology reasoning methods focus on knowledge representation and reasoning, facilitating better understanding and management of building data. The various algorithms class encompasses a diverse range of techniques including collaborative learning, adaptive Kalman filter (AKF), fuzzy logic, evolutionary algorithms, and more, each contributing uniquely to fault detection, pattern recognition, and system optimization. Collectively, these data-driven methods significantly improve the functionality, security, and efficiency of BMS, leading to smarter and more responsive building environments.

### 3.2. Classification of BMS Systems (RQ3)

**Figure 11:**
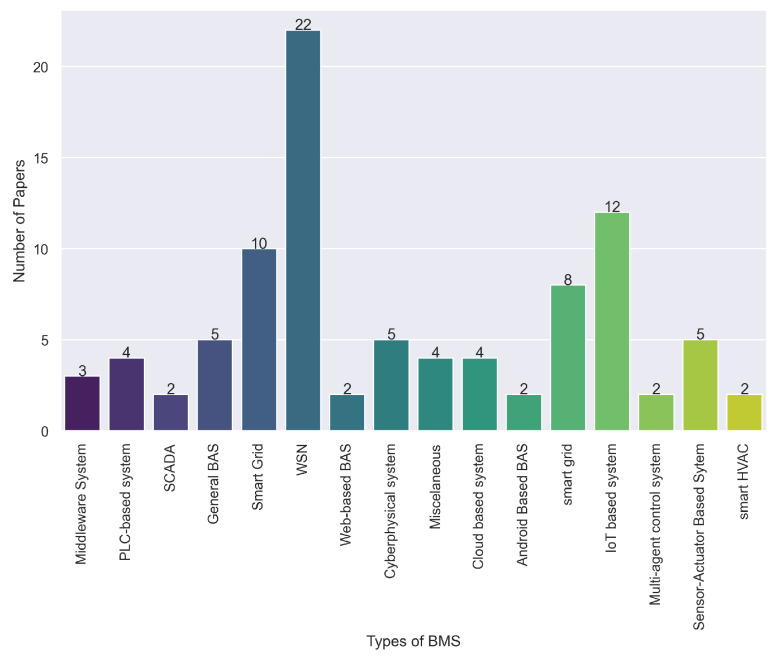
Number of papers employing each identified type of BMS.

RQ3 aims to identify and differentiate the variety of current BMSs and each unique and distinguishable feature. The term BMS itself encompasses all automation systems explicitly applied to buildings. However, recently there have been more devices controlling building elements. Consequently, primary studies have very diverse systems. To answer RQ3, we analyzed the authors’ description of their approaches and then compiled the classification as shown below. Additionally, we propose Table 5, which summarizes this section, and Figure 11, which graphically displays the predominance of each system in the primary studies.

***Middleware***

The term “Middleware” is often found in primary studies. This term describes a subsystem between services and field devices in a general way. The term middleware can be as general as BMS. However, some authors’ approaches conceive it as a type of BMS. In those approaches, the middleware system is presented as a disassociation from field devices and services as the logical and physical (sometimes only logical) bridge between them and only as. Its function is entirely dedicated to communicating the two levels by creating an abstraction that solves the complex wiring of field equipment. The reaching functionalities are often confused with the functionalities of a communication router or a multiplexor. However, the abstraction that contributes to the operation not only of communication but also with services remarks its key feature.

***Cyber-physical system***

Contrary to middleware systems, the key feature of cyber-physical systems (CPSs) has a more inclusive implication. Instead, CPS is an entire system that includes field devices, allowing the interaction between cyber and physical spheres. CPS is the conception of a complete, interconnected system comparable to a nervous system in the human body. Then, the key feature of a CPS is an interconnected network of sensors and actuators with a certain level of autonomy in each node.

***IoT-based system***

From the perspective of BMS’ composition, the primary studies refer to titles including “IOT-based system”. This refers to IoT as the main component of the system. IoT is defined as interconnected objects that allow the interaction between physical reality and the digital world [137]. The key feature of this system is the presence of multiple objects (such as sensors and actuators) connected to a local network or external network. An IoT-based system refers to a network-accessible system where users can retrieve the data directly from each IoT. Some IoT-based approaches work linked to building information modeling (BIM) platforms to complement and increase the precision of these systems. BIM-IoT approaches act as BMS limited to surveillance of the building.

***PLC-based system***

Programmable logical controllers (PLCs) or BMS PLC system, the main feature of which is the use of these robust devices as BMS, are based on the standard IEC 61131-3. These devices are mainly used in the industry as reliable control mechanisms, and some authors have used them to govern controllers in buildings. PLC-based BMS can execute tasks as a sequence of events a stimulus triggers. These systems usually visualize data in BMSic human–machine interfaces.

***SCADA systems***

A comprehensive system is called a supervisory control and data acquisition system (SCADA). This type of system is often found in buildings to centralize the supervision of sequences. They include a hub named Open Platform Communication (OPC) that merges all the data of sensors and actuators (usually, these systems use PLCs). Authors conceive SCADA systems as BMS for large buildings with many sub-spaces (e.g., hospitals and different sub-areas). Moreover, SCADA visualization features represent equipment in buildings, making a more comprehensive abstraction for users. SCADA systems are specified to supervise and provide direct control. Then, the key feature of this type of BMS is its centralized control and supervision of sequences of events.

***Smart grids***

Some researchers have conceived a system based on information from electric companies. Smart grid systems are based on the interaction of the building with the electric energy supplier. This communicates the energy pricing information to the building system. The smart grid systems identified in this SLR profit from the cost rate information supplied by energy companies via smart meters. The idea is to program the functioning high-consumption devices (e.g., boilers) at the most convenient hour. The key factor of this system is incorporating the supplying grid as part of the system, which leads to energy consumption.

***Wireless Sensor and Actuators Networks (WSAN)***

Buildings are intricate spaces where multiple systems (plumbing, illumination, security, etc.) co-work to enhance inhabitants’ lives. Installing these systems requires planning and understanding their life cycle and maintenance. This is also the case of buildings’ sensors and actuators systems. Researchers associate WSAN as a type of BMS with simplicity and inexpensiveness (plug-and-play philosophy).

WSAN’s key feature is its unique layout of wireless devices in a particular topology (mesh, tree, or start). The layout of WSAN includes intermediate gateway and routing nodes to make wireless communication more reliable.

***Multi-agent system***

Multi-agent systems have been proposed to control building elements and solve issues. The agents are a kind of autonomous device that possesses different knowledge. Then, they cooperate according to their capabilities and knowledge to solve common goals. These agents define their own behavior and communicate independently with each other. This type of system is used as a BMS system to improve the building’s energetic performance. Its key features are autonomous behavior and cross-communication between agents. Unlike other systems, communication is made between agents and not only to a hub.

***Smart HVAC***

The HVAC systems hardly impact energy distribution: in the European Union, buildings represent 40% of the total energy concern, whilst in the USA, buildings represent 70% of total energy consumption [50,60]. This is a solid motivation for researchers to optimize HVAC functioning, and some researchers associate BMS only with HVAC systems. The HVAC comprises subsystems such as heaters, ventilators, pumps, ducts, thermometers, and other sensors. HVAC’s primary goal is to orchestrate all its components to achieve a temperature level defined by a user. Researchers have proposed smart HVAC by adding a programming layer allowing users to schedule and target goals, as well as estimate the time to perform those changes.

***Sensor-based system***

Researchers conceive multiple specific applications. They name their approaches by only pointing to the sensors’ type and aim. Some examples might be “Passive Infra-Red (PIR) sensors-based BMS” or “Beacon-based BMS”. They might also refer to firmware such as “Android-based sensors for BMS system”. Sensors and their features drive this type of BMS.

***Cloud-based system***

Some authors move processing and decision making far from the data source. Cloud-based BMS retrieves data directly from sensors and actuators. Although it is common to find references to IoT in this type of system, the studies are centered on the functionalities and processing in the cloud. The key feature is the remote management of buildings, which is advantageous for handling multiple buildings (clusters).

***Web-based BMS***

Such diversified systems like BMS require complex integration. Some researchers focus on the diversity of sensors and actuators and their amount. Then, they propose platforms like BMSs that use URLs to conduct instructions and collect data from devices. This is the so-called Web of Things (WOT). This type of BMS centers data analysis in the cloud and delivers instructions and collection through APIs implemented in the devices.

***Generic BMS***

Some researchers do not attribute any naming or type to their BMS proposals. Instead, they refer to it as a general system applied to buildings.

***Miscellaneous***

Some BMS proposals do not follow a pattern and are independent innovations. Examples are multi-carrier hubs and organic systems.

The classification highlights each system’s key hallmarks. We are conscious of the possible ambiguity in defining each system type. For instance, CPS- and IoT-based systems have very similar key features. However, this is not the case. CPS is about complex interconnection, integration, and processing, while IoT-based systems connect objects and directly retrieve data. Beyond the SLR, there is some work as in [138] that addresses the difference between CPS and IoT. In this SLR, we confirm these differences, but not purposely. Indeed, when compiling each author’s description, their convergence confirms this. We made a similar analysis with other systems as in the case of CPS and IoT-based systems.

### 3.3. BMS-Dispersed Nature (RQ4)

**Figure 12:**
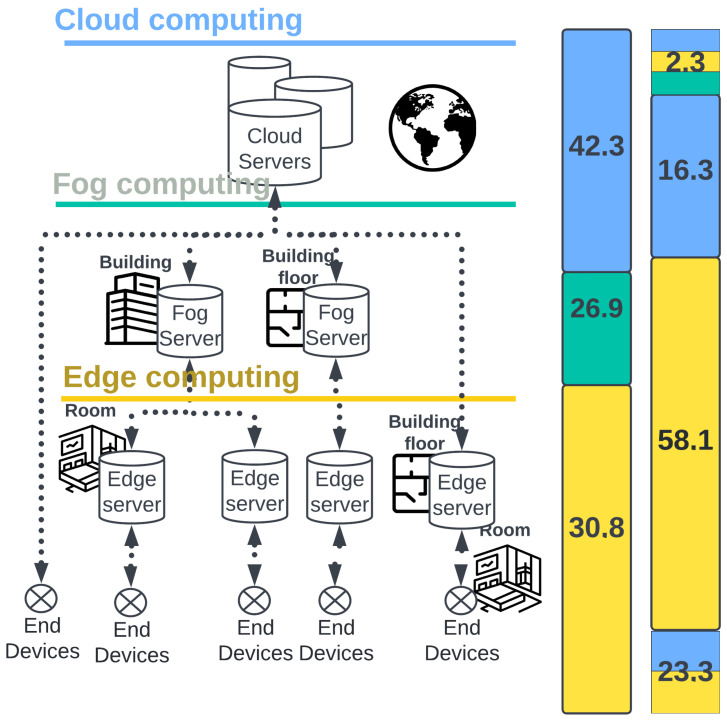
The different configurations of computing layers identified in the reviewed papers. The first column depicts in blue the percentage of Cloud computing papers, in green Fog, in yellow Edge. The second column shows four blocks describing papers that focus on Cloud and include the other layers (2.3%), only cloud (16.3%), Fog computing (58.1%), and Fog and Edge (23.3%).

RQ4 aims to identify how authors understand and deal with the BMS’ geographically dispersed nature. To solve RQ4, we have synthesized and categorized primary studies according to our pivoted definition of Cloud, Edge, and Fog computing. Since many papers still do not prefer this way of splitting BMS, we analyzed in each paper the type of computing (e.g., processing of data, storage, employment of algorithms, visualization, etc.) and where it is made (in the building or far from it). In this respect, the results are shown in Figure 12, which displays the percentage of papers according to the layers’ usages, and also shows where complex processing is made. Our findings are described as follows:

***Edge***

Edge computing has become an interesting research field due to its closeness to the data source. This SLR reports that most of the reviewed papers are centered in edge applications in around 58.1% [26,27,28,29,31,34,35,36,39,40,41,43,44,45,50,52,54,55,56,58,59,64,65,66,67,70,71,76,77,78,79,80,81,83,84,87,89,90,103].

***Fog***

While compiling the papers, we notice a mismatch between our pivoted definition given by [139,140] and the work of the author [74]. Since our mission is to summarize rather than debate, we excluded this paper from the analysis in this subsection. After this mismatch, we found no other approach to uniquely address this layer.

***Cloud***

We report that 5.8% of the reviewed papers [57,62,82,86,99] apply only cloud computing to manage buildings. We believe that this is a growing tendency, and more complex architectures incorporate these layers as part of their systems.

***Edge-Fog***

Systems at these two levels represent 10.5% [30,33,37,38,68,75,91,93,103], and their interaction is balanced. The processing in this type of layered system can involve computing at the edge level, such as data fusion, data transformation, and algorithmic processing, while at the fog level, they might host more complex algorithms and data visualization. In this case, data are processed in the edge devices, and information is transmitted to the fog level for visualization. Additionally, edge devices process simple tasks in other approaches, and data analytics are performed at the fog level. In this circumstance, the Fog applies more complex algorithms than the edge level.

***Edge-Cloud***

Combining these two layers is the most popular in complex architectures, and it represents 23.3% of the reviewed papers [31,32,42,46,47,48,51,53,56,72,73,74,85,92,96,100,102]. The interaction in this layer is more complex and richer than Edge-Fog systems. Cloud computing possesses more processing and storage capabilities and fewer maintenance constraints than any other layer. Like Edge–Fog systems, data are streamed from different sources to a central hub and simple operations are executed at the edge level. One of the key differences is the physical distance between the source and the data hub. Moreover, the augmented capabilities of the cloud allow the implementation of more complex algorithms and training models.

***Tiered architectures***

A more complex system scheduling tasks in these three layers represent our reviewed papers’ 2.3% [91,93]. This system is more complex and considers various tasks at each level. However, data and information management is different. The streaming of data from each source is gathered in a central local device. As Fog is an intermediate layer with more capabilities than the Edge to process data in the building, big data is shaped at this level. In this respect, we can describe the interactions in Edge–Fog as voluminous, quick, and diverse data streaming and Fog–Cloud as a synthesized exchange of data.

***Main processing***

The central processing in these complex architectures (Edge–Fog, Edge–Cloud, and Edge–Fog–Cloud systems) is most of the time made at the cloud level 42.3% [32,42,46,47,73,74,91,93], then Edge 30.8% [31,37,48,51,56,72,86,102], and an almost similar proportion Fog 26.9% [30,33,38,53,68,75,103] as shown in Figure 12.

We did not find a significant conflict between our pivoted definition and the authors’ approaches. However, we had a mismatch with the work of [74] that led us to exclude this paper only for this section. Since Fog computing’s formal definition is still unstable, it is comprehensible that some authors have a different vision of this layer.

### 3.4. Attribution of Smartness (RQ5)

There is incertitude behind the term “smart” and the underlying question of what makes something smart. We agree with the author [7] and its exhaustive effort to answer this question. This author equates smartness to a spectrum that describes 23 features that make something high or low smart. However, we believe that qualifying the smartness of the primary studies according to this definition might disrupt the authors’ self-perception. Additionally, the SLR aims to report rather than portray our and others’ perspectives. To solve RQ5, we collected the primary study authors’ attribution to smartness and classified them. In a few paragraphs, some authors explicitly address what they consider to be smart in buildings, and others do not attribute smartness to any feature. We found five noticeable patterns that the authors consider to be what makes a building smart, described as follows.

***Adaptability***

The authors in [39,43,45,47,56,65,69,71,76,77,78,82,87,89,90,91,94,105,127] contemplate the global system and how it can be adapted to new scenarios. In these studies, it is common to find terms such as reconfigurable systems, multiple dedicated services, self-configuration, autonomy, awareness, dynamic adaptation, retrofitting, and context-awareness. The understanding of these authors about what makes a building smart is a system that is flexible and able to change and evolve according to the users’ needs. Some of the proposals in this conception of smartness include open platforms that manage the multiple services dedicated to specific tasks. In short, what these authors believe makes a building smart is a system allowing this flexibility no matter the hardware devices or control algorithms.

***Decision-making algorithm***

Some authors [41,44,46,50,51,52,54,55,56,57,58,59,63,80,83,84,85,88,95,99,104] attribute the smartness of a building to the decision-making algorithm of BMS and the underlying equipment. Moreover, in the literature, keywords such as “in smart building” and “for smart buildings” are common. By using these keywords, the authors suggest that their approaches contribute to the smartness of an already smart building. These authors point out that algorithms such as learned and knowledge-based approaches largely contribute to the smartness of the building.

***Buildings’ equipment***

The authors [53,60,61,62,66,67,68,70,72,73,75,79,81,92,93,96,97,98,100,101,102,103,108,109,110] refer to BMS field equipment (sensors, controllers, and other devices) as the factor that becomes a building into smart. However, the authors do not mention any specific devices that make a building smart. Instead, they generally talk about an equipped building able to perceive and act accordingly.

***Processing data***

Processing refers to collecting, transforming, and creating new data [141]. Directed by this definition, the authors in [30,31,32,36,37,48,74,86] hold that any BMS with a processing capability transforms a building into a smart building. Following this understanding, we hypothesize that buildings with only controlling devices rather than collecting data might not qualify for this classification.

***Non-attributed***

In contrast to all the attributions explained before, some authors [27,28,29,31,33,34,35,38,40,42,106] even if they include the term smart system, they do not attribute smartness to any feature of a building or a system, nor opposed to the term smart. These contributions neglect the use of smart to define or highlight the advantages of their proposals.

**Figure 13:**
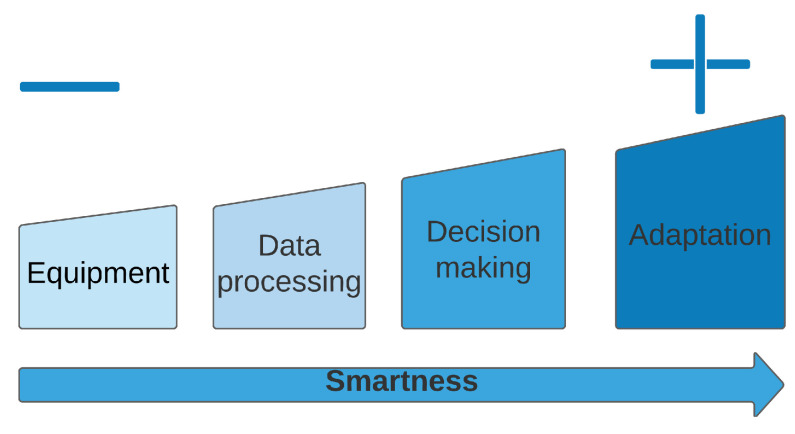
Conceptual framework of Section 3.5. The blocks are features of BMS that authors attribute as contributors to the smartness of a building. At the bottom, there is only BMS equipment, and on top, the accumulation of features (equipment + data processing + decision − making + adaptability).

The patterns identified in RQ5 validate the hypothesis of the spectrum of smartness. While compiling the primary studies, we did not find any evidence pointing towards isolated smart objects (e.g., smart doors) contributing to the smartness of a building. Instead, smartness is always related to an organized architecture with members directly or indirectly interacting, centralized or decentralized, as BMS does. Moreover, we identified a relationship between the variables (adaptability, decision-making algorithm, underlying equipment, and data processing). Following the hypothesis of the spectrum of smartness, we notice that there are more scientific bases to believe that adaptability is a very smart system feature. On the other hand, a building with only equipment seems to contribute less to the smartness. We propose what seems to be an interpretation of the compilation of this section. Figure 13 references how BMS features contribute to the smartness of buildings.

### 3.5. Summary of Research Questions and Key Findings

To provide a concise overview of the research questions addressed in this review and their corresponding key findings, Table 6 presents a summary that highlights the primary outcomes of each research question.

### 3.6. Research Directions (Reporting the Review)

This section synthesizes and lists the principal hurdles and research directions (RDs) identified in this SLR.

- **RD1:** *Dealing with spatial heterogeneity*

 This SLR highlights the many efforts of authors addressing the heterogeneity issue. However, there is a gap regarding the spatiotemporal aspects of buildings. It is a fact that descriptive explicit models are a need in this field. However, they should include more than functional and feature information BMS devices. There is a need to relate devices with their geographical position and spatial scope in a computable way. This need is scarcely addressed by researchers [31,32,38]. Some explicit models, such as Industrial Foundation Classes (IFC), seem to give some ideas of how IFC can relate devices and spatiality. However, IFC is a large meta-model conceived to achieve interoperability in the construction field. Consequently, some parts of the IFC meta-model might not benefit BMS. Some other explicit models are BRICK schema and Haystack, which provide a meta-model to objects, yet the relation of buildings’ spatiality is incomplete.
 Moreover, information about devices’ processing and storage capabilities is missing. Since devices are no longer endpoints with limited functions, we believe that the evolution of the explicit models must follow the evolution of the devices. Having this information will be fundamental for further development. Hence, distributing BMS’ resources to solve complex issues is achievable.

- **RD2:** *Exploiting structured and unstructured data*

 Many solutions to the heterogeneity of data and devices include explicit models to structure this information. These models provide rich context to data, making, for example, learning-based model more accurate. This is reflected in the reviewed papers, for example, in [79], where microphone sensors are used to identify the spaces’ occupancy and purpose, to manage the heating of the HVAC system. Still, it is scarcely explored by authors. The type of structuring model advantage seems proportional to the explicit model type; for instance, some authors have employed BIM to describe building topology, to providing data a 3D visual background.
 It is advisable, that further research focus on using and combining high order semantic models, such as ontologies, to structure, refine, and control the automatic decision-making of BMS subsystems.

- **RD3:** *Defining layers’ roles*

 In most papers, the researchers focus on a particular layer. Solely a few authors address the roles of each layer in BMS’s tiered architectures [91,93]. Indeed, no substantial work exists on each computing layer’s (Cloud, Fog, and Edge) role and in relation with others. In [91], the capabilities of each layer define its roles and interaction. Notice that, in this approach, BMS works hierarchically. In this case, the most capable layer holds the decision-making algorithm. However, other aspects, such as security, privacy, and efficiency, should also be considered and explicitly justified.
 The authors address multiple decision-making layers as a multi-agent-based BMS. In this case, each agent can act and collaborate autonomously. However, there is unknown work in hierarchical collaborative systems. We did not find any work addressing this gap in this SLR, yet almost all tiered architectures work in hierarchies. Creating a collaborative environment in two—or three-tiered architectures seems to be an emerging research field. The need for federated solutions to solve possible conflicts, building response time, seems to be evident. Otherwise, it would be risky for occupants to operate BMS with a non-reasonable meaning.

- **RD4:** *Safety on occupants’ data*

 The close interaction between BMS and the dwellers requires privacy and security policies to ensure this information. The flow of information in buildings has two directions from the source (e.g., occupancy) and towards the source (e.g., remote controlling). The author [64] addresses the security of BMS, identifying attack types such as changing systems’ set points, the falsification of sensors’ measurements and control signals, and the modifications of commands. However, privacy aspects are only covered in one direction (from the source).
 An emerging field and further research in the building field seems to be the study of privacy in a geographically dispersed environment. Since there is more concern about where data are stored, some governmental institutions such as the European Union (EU) are starting to regulate this.

- **RD5:** *Improving Operativity of BMS*

 This SLR identifies many innovative services to solve use cases. However, very little has been said about the operativity of BMS. Since many authors focus on the acquisition and exploitation of data, the operativity of BMS is seldom addressed. Some work [93] addresses the communication between devices by applying algorithms like Robin Round. Some others, like [71], work on anomaly detection in BMS devices. In this respect, we believe there should be more research on the preventive maintenance of BMS devices.

## 4. Conclusions

This SLR summarizes the literature of the last decade around building systems from a computer science view. The aim is to summarize cutting-edge development in BMS and provide framework development to solve RQs. Additionally, we report our findings as research directives concerning fields of BMS where research is demanded. We highlight the main findings as follows:

 **There are eleven fields of interest around BMS**
 We could see that the researchers’ interests are divided in the identification. Some researchers address occupants’ needs and work on how BMS can improve their well-being. From these studies, the field most addressed is the reduction in energy consumption in buildings. On the other hand, some other researchers address BMS issues. These studies focus more on solving data heterogeneity.

 
**Decision making is mainly made by five types of algorithms**
 Fuzzy logic, knowledge bases, and learning-based algorithms. Additionally, we also found horizontal development that aims to enable data across different subsystems using knowledge bases and ontologies.

 
**There are 14 types of BMS**
 We identified innovative solutions using current technologies such as IoT, CPS, and Web solutions.

 
**The BMS is a two- or three-layered architecture**
 The SLR has identified that, among computing types, BMS has three types: Edge, Cloud, and Fog computing. The researchers have a noticeable preference for Edge development. It was found that 58.1% of papers report development at the Edge, followed by tiered systems (36.1%) and Cloud (5.8%). The authors distribute the task load principally in the tiered architectures in the Cloud. At around 42.3%, the central processing is made in the Cloud, followed by Edge (30.8%) and Fog (26.9%).

 
**Smartness is linked to BMS features**
 Smartness is linked to the following BMS features: system adaptability, the decision-making algorithm, the underlying equipment, and data processing.

This SLR illustrates the transition from single-minded solutions to integrated frameworks that aim to combine data and supervision within a single subsystem. This transformation turns buildings into entities capable of reasoning, learning, and adapting accordingly.

## Figures and Tables

**Table 1:** Table containing the principal and auxiliary sources of information. The subject of the library is CS for (computer science), M (multidisciplinary), and EEE (electrical and electronic engineering).

Library	Open Access	N Boolean	Subject
Principal			
Google Scholar	Yes	6	M
IEEE Xplore	Non	7	EEE
DBPL	Yes	4	CS
Auxiliar			
ACM Digital Library	Yes	5	CS
Science Direct	Yes	8	M
Scopus	Non	8	M
Springer Link	Non	8	M
Web of Science	Non	9	M
Wiley Online Library	Non	9	M

**Table 2:** Quality criteria questions to display the quality of the papers.

Quality Criteria
QC1: Do the authors explicitly state the motivation behind their research, and is it relevant to the field of BMS?
QC2: Are the approaches described enough?
QC3: Is the contribution of the paper shown explicitly with enough details?
QC4: Do the authors discuss the achievements and limitations of the research and the approaches themselves?

**Table 3:** Summary of findings in RQ1. In services, the algorithms are classified in: knowledge bases (KBs), learning-based algorithms (LBA), open platforms (OP), fuzzy logic (FL), and various algorithms (VAs).

BMS-Service-Oriented Fields		Identified Issue	Proposed Solutions	Authors Addressing These Fields
Energy consumption	Occupant behavior	Identifying the occupants’ waste patterns	Predict occupants’ behavior accurately	VA [29,31,59,66,72,78]; LBA [112] FL [60]
	Localization and occupancy	Identifying the purposes of buildings’ spaces	Classifying spaces by their designated purpose and properties	KB [63,65,67,71,74,77,79,80,81,89,90,91,105,106,107] LBA [113,114,115]
	Demand-based solutions	Handling different energy sources	Complementing the pricing information with thermal and scheduling models	VA [32,49,55,75,97,99,102] KB [50,85]; LBA [56,116,117,118],
Healthcare		Self-awareness and privacy aspects	Applying learning-based algorithms and anonymization of data	VA [42]; LBA [43]
Indoor navigation		Constructing pathways and distributing this information	Adding contextual information of the occupancy of rooms	VA [54,76,84,92,101,110] LBA [87]
Occupants well-being	Air quality	Determining the dispersion of gases	Applying learning-based algorithms to estimate the dispersion	LBA [57]; VA [98]
	Thermal comfort	Finding the best combination of events to trigger HVAC	Adding context to data (e.g., size of rooms)	LBA [53,119]
	Illumination	Adapting thresholds to the seasonal changes	Dealing uncertainty of changes by adding multicriteria decision rules	FL [108]
Multipurpose applications		Enhancement of BMS	Applying explicit models, and open platforms as system information.	OP [45,68,86]; KB [82] LBA [64]; VA [109]
**BMS-architecture-oriented fields**				
Heterogeneity of the data source	Data heterogeneity	Dealing with the variety of data	Applying explicit models to structure data	[5,26,27,28,35,36,62,120,121,122,123,124]
	Devices heterogeneity	Dealing with the diversity of devices’ features		
Big data management		Handling velocity, volume of data, the scalability of databases, and parallel processing in a dispersed environment	Applying parallel processing, non-relational database, and performing data wrangling	[30,34,39,40,52,57,58,63,71,72,83,94,96,102,125]
Decision-making-related issues		Dealing with the execution of BMS actions	Applying learning-based algorithms, knowledge bases, fuzzy logic, various algorithms	[31,32,37,38,41,47,59,61,100,126,127]
Adaptability		Creating horizontal development	Customizing existing operating systems (e.g., Android)	[42,44,45,46,60,74,78,80,90,93,95,101,112,128,129,130,131]

**Table 4:** The data-driven methods identified in RQ1.

Class	Method	References
Machine Learning	Anomaly Detection (Isolation Forest)	[64,126]
	Artificial Neural Network (ANN)	[48,57,112,113,117,119]
	Ensemble Learning with Various Techniques	[125]
	Explainable AI (SHAP)	[132]
	Hybrid Machine Learning Predictive Models	[123]
	k-Nearest Neighbor (k-NN)	[114]
	Linear Discriminant Analysis (LDA) for Offline Learning	[91]
	Reinforcement Learning (RL)	[116,117]
	Occupant Behavioral Models	
	Radial Basis Function Network (RBF)	[87]
	Support Vector Machines (SVMs)	[54]
	Unsupervised Learning	[133]
Deep Learning	CNN and LSTM Models	[118,129,134]
	Faster R-CNN Model	[115]
	Various Architectures (GANs, VAEs, RNNs)	[131]
	Faster Region-based Convolutional Neural Network (R-CNN)	[115]
	Deep Reinforcement Learning (RL)	[128]
Semantics and Ontology Reasoning	Knowledge Representation and Reasoning	[120,121,124,135,136]
Various Algorithms	Collaborative Learning and Virtual Organizations of Agents	[59]
	Face Detection, Expression Recognition, and People Trackers	[90]
	Complex Event Processing (CEP)	[76]
	Adaptive Kalman Filter (AKF)	[71,109]
	Fuzzy Logic	[79]
	Cooperative Bargaining Game Model	[67]
	Hybrid Bacterial Foraging and Genetic Algorithm (HBG)	[72]
	Evolutionary Algorithm (EA)	[75]
	Fuzzy Self-Tuning Particle Swarm Optimization (FST-PSO)	[118]
	Simulated Annealing	[97]
	Min-Min Algorithm	[102]
	Pattern Detection and Data Analysis	[53]
	Hidden Markov Model (HMM)	[81,84]
	Minority Game (MG) Algorithm	[85]

**Table 5:** List of types of BMS found in the primary studies.

Type of BMS	Key Feature	References
Middleware system	Abstraction of field devices	[26,27,28]
CPS	Interconnected network with autonomy of nodes	[36,44,77,81,100,107]
IoT-based system	Interconnection of nodes through and internal or external networks	[47,60,63,67,73,76,82,83,91,94,98,101,104,108,109]
PLC-based system	Robust and autonomous nodes	[29,37,41,51]
SCADA	Centralized control and supervision	[30,54]
Smart grids	Energetic pricing-driven systems	[32,46,56,68,71,84,92,95,99,102]
WSAN	Wireless network with a specific topology	[33,38,53,58,59,61,62,65,69,70,80,86,88,110]
Multi-agent system	Self-driven agents	[48,64,103,105]
Smart HVAC	Thermal-comfort-based BMS with programmable scheduling	[52,97]
Sensor-based system	Sensor-based specific approaches	[50,66,74,75,78,87]
Cloud-based system	High processing and store capabilities far from the source of data	[40,57,85,89]
Web-based system	Light API’s on devices to retrieve and deliver data	[35,42]
Generic BMS	General features of BMS (sensors and controllers)	[31,34,55,56,79,106]

**Table 6:** Research questions and key findings.

Research Question	Key Findings
RQ1: What are the prevailing technological and operational interests and services in the BMS field?	- Integration of IoT and AI for enhanced building management. - Focus on energy efficiency and sustainability. - Development of user-centric BMS interfaces.
RQ2: What methods are employed to analyze and utilize data in BMS to enhance operational services?	- Use of machine learning techniques. - Real-time data analytics for predictive maintenance. - Implementation of cloud computing for data storage and analysis.
RQ3: What are the different types of BMS available, and what are their defining features?	- Centralized vs. decentralized systems. - Features include scalability, interoperability, and user-friendliness.
RQ4: How is computing architecture distributed within BMS across different geographical settings?	- Variation in architecture. - Adoption of edge computing to reduce latency. - Hybrid architectures combining cloud and local servers.
RQ5: How do BMS contribute to the smartness of buildings, and what specific features are most impactful?	- Automation of building operations. - Enhanced energy management leading to cost savings. - Improved indoor environmental quality (IEQ) through smart sensors.

## Data Availability

The original contributions presented in the study are included in the article/Supplementary Materials, further inquiries can be directed to the corresponding author.

## Supplementary Materials

The following supporting information can be downloaded at: https://www.mdpi.com/article/10.3390/s24134405/s1, Supplementary S1: PRISMA 2020 Checklist; Supplementary S2: PRISMA 2020 flow diagram for new systematic reviews which included searches of databases and registers only. Reference [142] is cited in the Supplementary Materials.
